# Revision of the comose flame moths of the genus *Sosxetra* Walker (Noctuidae, Dyopsinae), with descriptions of a new genus and three new species

**DOI:** 10.3897/zookeys.1268.138260

**Published:** 2026-02-06

**Authors:** Jose I. Martinez, Nicholas T. Homziak, Taylor L. Pierson, Rhys J. L. Campo, David M. Plotkin, Raiza J. Castillo-Argaez

**Affiliations:** 1 McGuire Center for Lepidoptera and Biodiversity, Florida Museum of Natural History, University of Florida, Gainesville, FL, 32611, USA Florida Museum of Natural History, University of Florida Gainesville United States of America; 2 Florida State Collection of Arthropods, Florida Department of Agriculture and Consumer Services, Division of Plant Industry, Gainesville, FL, 32608, USA Department of Agronomy, University of Florida Gainesville United States of America; 3 Department of Agronomy, University of Florida, Gainesville, FL, 32611, USA Florida Department of Agriculture and Consumer Services, Division of Plant Industry Gainesville United States of America

**Keywords:** Barcoding, cryptic species, Lepidoptera, morphology, neotropics, systematics, taxonomy

## Abstract

As part of our Neotropical Dyopsinae Project, a revision of the Neotropical genus *Sosxetra* Walker is proposed. Morphological and molecular evidence challenge its previous monotypic classification. The genus is redescribed and a neotype is designated for *Sosxetra grata* Walker, previously considered the only species in the genus. Two new species are described: *Sosxetra mamanina* Martinez, Homziak, & Castillo-Argaez, **sp. nov**. and *Sosxetra thutakanay* Martinez, Homziak, & Castillo-Argaez, **sp. nov**. Finally, *Covellana* Martinez, Homziak, Plotkin & Castillo-Argaez, **gen. nov**., is established based on *Covellana niomalan* Martinez, Homziak, Plotkin & Castillo-Argaez, **sp. nov**., previously misidentified as *Sosxetra grata*.

## Introduction

The *Ceroctena* clade was initially proposed by Zahiri et al. (2013), but it included the genera *Belciana* Walker and *Cyclodes* Guenée, which were later demonstrated to form distinct clades by Martinez (2022). Here, the *Ceroctena* clade, termed the comose moths (Lepidoptera: Noctuidae: Dyopsinae), is composed of the genera *Ceroctena* Guenée, *Covellana* Martinez, Homziak, Plotkin & Castillo-Argaez, gen. nov., *Pachyplastis* Felder, and *Sosxetra* Walker, and the *Desmoloma* Felder and *Ortopla* Walker generic complexes (Keegan et al. 2021; Martinez 2022). For decades, the taxa in this clade were placed either in the erebid subfamily Lymantriinae (tussock moths) or Calpinae (Poole 1989), but they are currently all placed in subfamily Dyopsinae (Zahiri et al. 2013; Keegan et al. 2021; Martinez 2022). Most species in this clade resemble tussock moths, particularly in their resting position. We chose “comose flame moths” as the common name for this clade, deriving “comose” from the Latin word “*comosus*”, meaning “hairy”, to reflect their resemblance to tussock moths (Lymantriinae). The word “flame” was selected to evoke the vivid reddish-orange coloration commonly seen on the wings of many species within this group.

The genus *Sosxetra* Walker was originally described as a monotypic genus. The type species, *Sosxetra grata* Walker is only known from a brief description (Walker 1862) and the type specimen is presumably lost, as there is no information available regarding its depository institution (Poole 1989). Here, we designate a neotype and redescribe this species. We also describe two new species: *Sosxetra mamanina* sp. nov. and *Sosxetra thutakanay* sp. nov. Additionally, we describe a new genus in the *Ceroctena* clade, *Covellana* gen. nov., with a new species, *Covellana niomalan* sp. nov.

## Materials and methods

Terminology used for describing genitalic morphology, and methods used for genitalia dissection and preparation, follow those of Lafontaine (1987, 2004) and Schmidt and Anweiler (2020). Genitalia were stained with 10% eosin Y and examined in 30% ethanol. Genitalia preparations of type specimens were slide mounted with Euparal. Preparations of other specimens were stored in microcentrifuge tubes with glycerin. We photographed the adults prior to dissection, employing a StackShot automated focus stacking macro rail with a Canon EOS 7D camera and a Canon EF-S 60 mm f/2.8 USM Macro lens. Genitalia were photographed after mounting using a different StackShot system equipped with a Canon EOS 6D camera and an Infinity long-distance microscope lens Model K2 DistaMax™. Photographs are included either under a Creative Commons Attribution-NonCommercial 4.0 International License (CC BY-NC 4.0) or with written permission from the photographers.

To molecularly identify the specimens, mitochondrial DNA (mtDNA) was used to generate species-specific DNA barcodes. DNA barcoding was conducted using a fragment of the mitochondrial cytochrome c oxidase subunit I (COI) gene for six specimens of *Sosxetra*, including one specimen of *S. grata*, and one specimen of *C. niomalan*, which were subsequently submitted to GenBank (accession numbers: PQ406478–PQ406483, PV077143) (https://www.ncbi.nlm.nih.gov/genbank/). Additionally, we used 25 ingroup specimens: 22 of *Sosxetra* and 3 of *Covellana*. It is important to highlight that, of the 136 DNA barcodes (127 publicly available) of *Sosxetra* deposited in the Barcode of Life Data System v. 4 (https://v4.boldsystems.org/), 118 specimens belong to *S. grata*, primarily from Costa Rica (82 specimens), with fewer samples from Honduras (1 specimen), Mexico (4 specimens), Panama (10 specimens), Argentina (2 specimens), French Guiana (9 specimens), and Peru (11 specimens). However, we included only those barcodes that matched our sequences with 100% identity and few barcodes from Costa Rica (17 specimens). In contrast, all available sequences for the other *Sosxetra* species were included: *S. mamanina* (4 specimens) and *S. thutakanay* (5 specimens). Finally, we incorporated the three available sequences of *C. niomalan*, which show up to 0.2% divergence.

**Figure 1.**
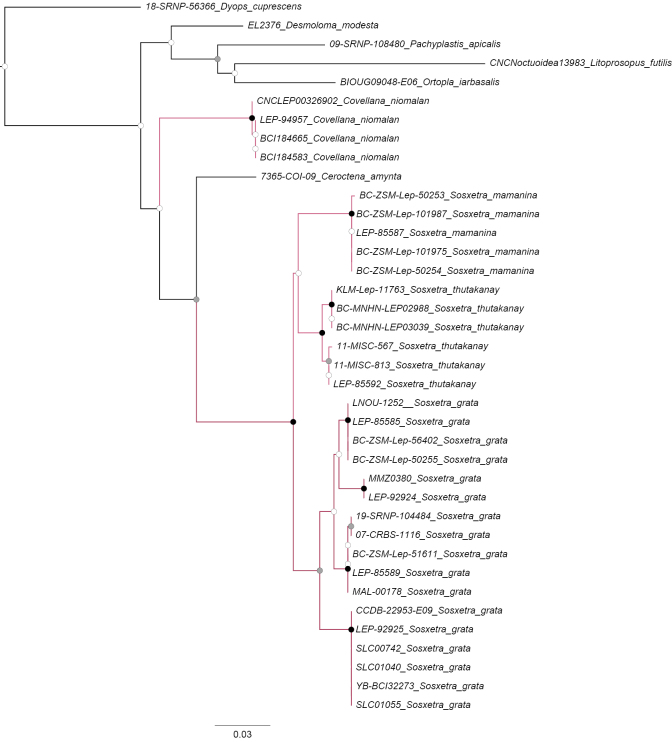
A maximum likelihood gene tree illustrating the relationships between the *Sosxetra* genus and other comose moth genera, based on the cytochrome c oxidase I gene (COI) marker. Nodes with black circles represent high support (UFBoot ≥ 95 and SH-aLRT ≥ 80). Nodes with gray circles represent medium support (UFBoot < 95 or SH-aLRT < 80). Nodes with white circles represent low support (UFBoot < 95 and SH-aLRT < 80). Black branches constitute the outgroups.

Four outgroup species were selected from the Barcode of Life Data System v. 4 (https://v4.boldsystems.org/) representing the rest of the comose moths genera (*Ceroctena* Guenée, *Desmoloma* Felder, *Ortopla* Walker, and *Pachyplastis* Felder), and two other two genera of dyopsines (*Dyops* Guenée and *Litoprosopus* Grote). DNA extractions were performed using legs and abdominal tissue removed from pinned dry specimens. Sanger sequencing was performed by Eurofins Genomics LLC, Louisville, Kentucky (https://eurofinsgenomics.com/en/home/). The sequences were concatenated and aligned using Geneious 9.1.3 (https://www.geneious.com/). The gene trees were constructed using a maximum-likelihood (ML) analysis performed in IQ-TREE v. 2.1.0 to establish relationships among taxa following protocols of Nguyen et al. (2015), Hoang et al. (2018), and Minh et al. (2020). We estimated branch support by performing 1000 replicates each for both ultrafast bootstraps (UFBoot2) (‘-bb’ comand) and the SH-aLRT test (SH-aLRT) (‘-alrt’ comand). Nodes were considered to have strong phylogenetic support if UFBoot ≥ 95 and SH-aLRT ≥ 80 (Fig. 1, Suppl. materials 1, 3). Molecular data were submitted to GenBank (https://www.ncbi.nlm.nih.gov/genbank/); accession numbers are listed in Suppl. material 2.

Data on the larval stages of *Sosxetra grata* were collected from 23 larvae by the senior author in Sacatepéquez, Guatemala, feeding on *Guarea luxii* C. DC (Meliaceae). Of these, only seven individuals completed the full larval development, although none successfully pupated. As a result, critical aspects of the life cycle of this species, remain undocumented. Further research is essential to fully characterize the complete development of *S. grata*, with particular emphasis on early instar stages, pupation, and host plant associations.

Specimens were obtained from the following museums and collections:

**JIM** Research Collection of Jose I. Martinez, Gainesville, Florida, USA.

**MfNB** Museum für Naturkunde Berlin, Berlin, DEU.

**MGCL** McGuire Center for Lepidoptera and Biodiversity, Florida Museum of Natural History, Gainesville, Florida, USA.

**MPM** Milwaukee Public Museum, Milwaukee, Wisconsin, USA.

Distribution maps were created using data from both the examined specimens and the barcoded specimens included in the Barcode of Life Data System v. 4 (https://v4.boldsystems.org/), and were generated via the publicly available website SimpleMappr (https://www.simplemappr.net/#tabs=0).

## Systematics

Implementing morphological and molecular data in our revision of Neotropical dyopsines, we found that the monotypic genus *Sosxetra* is in fact a complex of species and genera. The supposed phenotypic variations in *Sosxetra grata* are now shown to be characters that classify them into two genera and multiple species, as determined here:

1. pale lineage (*Sosxetra*): Composed of the type species of *Sosxetra*, *Sosxetra grata* and two new species *S. mamanina* sp. nov. and *S. thutakanay* sp. nov. These species can be distinguished from the other group primarily by the bright coloration of males and by the structure of the male antennal flagellum, which is bipectinate in the proximal ½ and filiform distally; male forewing with wide white reniform spot and well-developed medial and postmedial lines in cream or different tones of white; hindwing with posterior fringe in different tones of yellow; rectangular fused valvae with pointed apices; aedeagus and vesica simple; anal papilla remarkably wide relative to the abdomen; rectangular antevaginal plate; ductus bursae slightly sclerotized; corpus bursae and appendix bursae sclerotized.
2. dark lineage (*Covellana* gen. nov.): Restricted to the type *Covellana niomalan* sp. nov., in which the males exhibit a notably darker coloration compared to those of the other lineage; antenna is bipectinate except for the proximal and distal ends, which bit lack pectinations; male forewing with wide black curved line and black postmedial and subterminal lines well-defined; hindwing with posterior fringe in different tones of brown; Dutch clog-like (wooden footwear) valvae with rounded apex; aedeagus and vesica presenting diverticula. Female genitalia unknown.

### Key to the genera of the *Sosxetra* genus group based on male morphology

**Table:** 

1	Forewing with white reniform spot; transverse lines in different tones of white; rectangular valva with pointed apex; aedeagus and vesica simple (Figs 2, 3, 5–15, 18–20)	*** Sosxetra ***
–	Forewing with dark curved reniform spot; transversal lines in black; mezzaluna-like valva with rounded apex; aedeagus and vesica with diverticula (Figs 4, 16, 17, 21)	*** Covellana ***

#### Sosxetra

Taxon classificationAnimaliaHymenopteraEurytomidae

Walker, 1862

58BB36BC-4041-595B-BE23-198928C16688

Chaetoloma actinobola Felder, 1874: pl. 99, fig. 20 (syn. Poole 1989).
Ceroctena Möschler, 1880 (nec. Guenée, 1852): 469–470, pl. 9, fig. 21 (syn. Poole 1989).

##### Gender.

Feminine.

**Figures 2–4.**
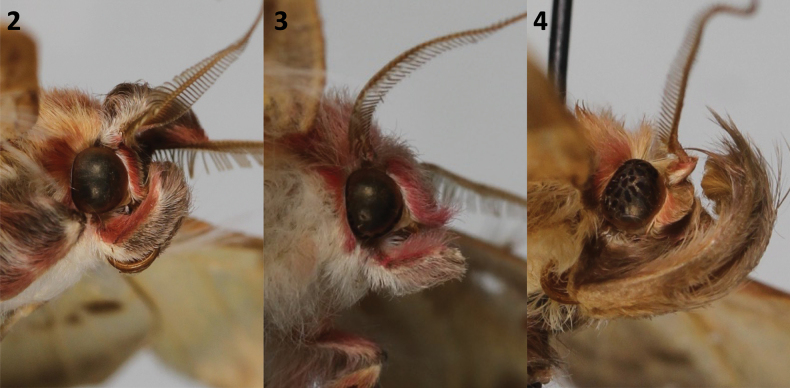
Adult head structure. **2**. *Sosxetra grata*, ♂, Yucatan, Mexico, MGCL; **3**. *S. grata*, ♀, Alajuela, Costa Rica, MGCL; **4**. *Covellana niomalan*, Paratype, ♂, Madre de Dios, Peru, MGCL.

**Figures 5–17.**
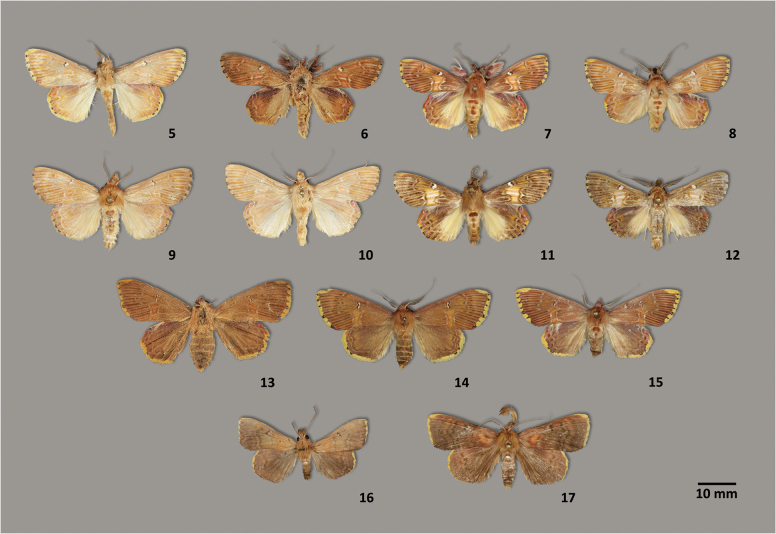
Adult habitus of *Sosxetra* and *Covellana*. **5**. *Sosxetra grata*, ♂, neotype, Espirito Santo, Brazil, MPM; **6**. *S. grata*, syntype of *S. agatha*, ♂, Suriname, MfN; **7**. *S. grata*, ♂, Napo, Ecuador, MGCL; **8**. *S. grata*, ♂, Yucatan, Mexico, MGCL; **9**. *S. mamanina*, ♂, holotype, Madre de Dios, Peru, MGCL; **10**. *S. mamanina*, ♂, paratype, Madre de Dios, Peru, MGCL; **11**. *S. thutakanay*, ♂, holotype, Napo, Ecuador, MGCL; **12**. *S. thutakanay*, ♂, paratype, French Guiana, MGCL; **13**. *S. grata*, syntype of *S. agatha*, ♀, Suriname, MfN; **14**. *S. grata*, ♀, Pichincha, Ecuador, MGCL; **15**. *S. grata*, ♀, paratype, Alajuela, Costa Rica, MGCL; **16**. *Covellana niomalan*, ♂, holotype, Canal Zone, Panama, MGCL; **17**. *C. niomalan*, ♂, paratype, Madre de Dios, Peru, MGCL.

**Figures 18–22.**
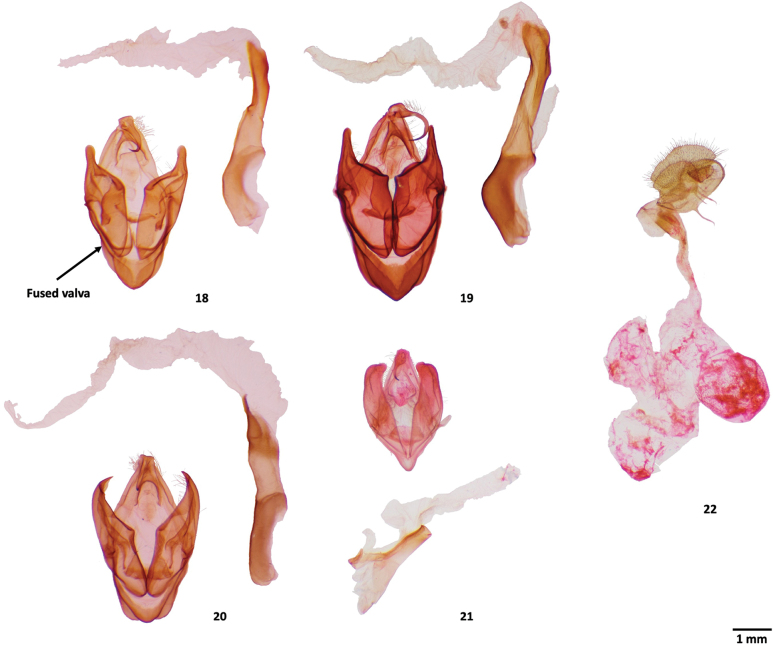
Male genitalia of *Sosxetra* species. **18**. *Sosxetra grata*, neotype, Espirito Santo, Brazil, MPM; **19**. *Sosxetra mamanina*, holotype, Madre de Dios, Peru, MGCL; **20**. *S. thutakanay*, ♂, holotype, Napo, Ecuador, MGCL; **21**. *Covellana niomalan*, ♂, paratype, Canal Zone, Panama, MGCL; **22**. *S. grata*, ♀, Alajuela, Costa Rica, MGCL.

##### Type species.

*Sosxetra grata* Walker, 1862, original designation by monotypy. Characters of undescribed Lepidoptera in the collection of W. W. Saunders, Esq., F. R. S. Transactions of the Entomological Society of London 3(1): 80.

##### Included species.

*Sosxetra* was described as a monotypic genus, containing only *S. grata*. The species *S. actinobola* and *S. agatha* were subsequently synonymized within it by Poole (1989). Additionally, we were able to identify two new species which we describe in the present study: *S. mamanina* sp. nov. and *S. thutakanay* sp. nov.

##### Diagnosis.

*Sosxetra* closely resembles *Covellana* externally. *Sosxetra* has a reddish-pink or brownish-red body, similar to *Covellana*, while *Ceroctena* displays various shades of green. Like *Ceroctena* and *Covellana*, the antenna is bipectinate but differing by the lack of pectinations in the distal ¹/3 of the antenna. The interfacetal setae of *Sosxetra* are short, as in other dyopsines. Male genitalia present fused valvae through the base of sacculus, which are impossible to separate without damage and similar to those of *Ceroctena* and *Covellana*. The valva is wide and heavy with a pointed apex differing from that of *Covellana*, which has a rounded apex. *Sosxetra* has a phallus larger than *Ceroctena* and *Covellana* with the opening to the vesica narrower. Female genitalia have very wide anal papillae densely covered by long setae like in *Ceroctena*. The corpus bursae is remarkably wider than in *Ceroctena*.

##### Description.

***Head***. Palpus sexually dimorphic, longer and wider in male; palpus usually marbled with brown, whitish-brown, and pink or red; frons concolorous with thorax; antenna with basal ²/3 bipectinate, apical ¹/3 filiform, female with shorter pectinations; antennal scape with long tufts of scales concolorous with palpus, tufts absent in female. ***Thorax***. Dorsum darker laterally and paler medially, ventrum whitish-brown; patagium concolorous with thorax, but whitish-brown margins. ***Leg***. Whitish-brown; foreleg with long tufts marbled brown, whitish-brown, and pink to red. ***Wing***. Forewing length 35–43 mm; ground color reddish-pink to brownish-red with whitish-brown lines edged dark brown; medial and postmedial lines distinct, others faint; area between medial and postmedial bright orange or pink; veins darkened; interveinal spaces paler; orbicular spot obscure; reniform spot S-shaped, white, bordered dark brown; terminal dashes black with purplish-blue scales; hindwing similar coloration; postmedial line distinct; discal spot faint whitish-brown; subterminal shade purplish-pink with purplish-blue line; terminal line yellow with three black dashes (M1–CuA1); costal margin with long whitish-brown hair-like scales; purplish-pink costal mid-lump; fringe brownish-yellow; ventral side whitish-brown with black shading; female darker; fringes bright yellow; hindwing posterior fringe variable yellow. ***Abdomen***. Dorsum brownish-yellow; tufts concolorous with thorax; ventrum side whitish-brown. ***Male genitalia***. Valvae fused, rectangle-shaped; apex pointed; tegumen narrow; juxta linear; uncus long, claw-shaped; saccus arrowhead-like; phallus short, narrow, elongate; vesica long, equal in length to aedeagus. ***Female genitalia***. Antevaginal plate broad, rectangular; sterigma narrow; ductus bursae slightly sclerotized; corpus and appendix bursae sclerotized; corpus bursae without signum; apophyses anterior and posterior subequal.

##### Genetic characterization.

Our molecular analysis indicates that *Sosxetra* is sister to the genus *Ceroctena* diverging by ≥ 5.6% (Fig. 1), which is consistent with other characters such as external morphology (Fig. 1).

##### Etymology.

Unknown.

##### Immature stages.

**Figure 23.**
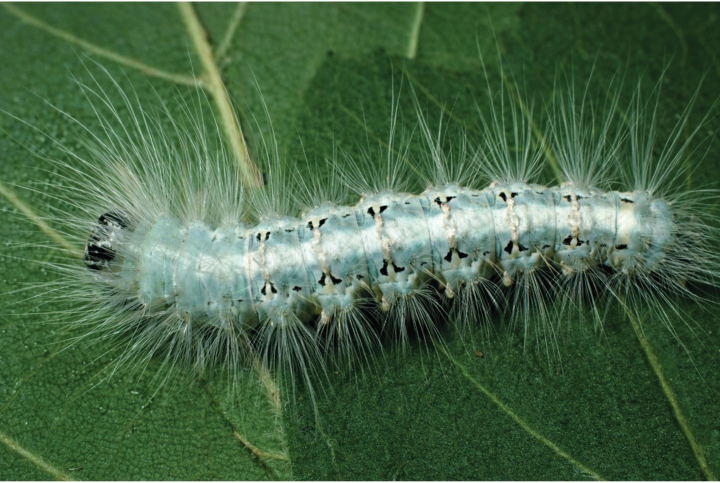
Last larval instar of *Sosxetra grata*, Guanacaste, Costa Rica, photo by Daniel H. Janzen.

***Egg***. Unknown. ***Larva***. Late instar with pale green or greenish-yellow color; head with black and white, yellow, or orange patterns; integument appearing greasy, similar to other dyopsines; two broken black subdorsal lines surrounding white or yellow dots with a blurry white line below them; a pair of pale gray, yellow, or cream dorsal hardened plates in every tergite from A2-A7; wide verrucae with long hair-like setae; wide whitish-green or whitish-yellow dorsal band (see *Sosxetra grata*, immature stages) (Fig. 23). ***Pre-pupa***. Integument changes to a dark reddish-pink or bright brownish-orange; subdorsal line changes to white or yellow subdorsal spots; dorsal brown or dark brown band A2-A7 including the area between the subdorsal spots; hardened plate darker than late instar; verrucae with long white or dark gray hair-like setae. ***Pupa and cocoon***. Unknown.

##### Biology.

*Sosxetra grata* is the only species in the genus with recorded life history. The larvae feed on species of the genera *Guarea* F. Allam. and *Trichilia* P. Browne (Meliaceae) (Janzen and Hallwachs 2009; JIM, per. obs.). The adults are diurnal and are usually observed puddling on leaf litter, mud, or tree bark, although they can also be attracted to artificial light (DH Janzen per. comm. 10/03/2023; JIM, per. obs.).

### Key to species of the genus *Sosxetra* based on adult male morphology

**Table:** 

1	Forewing with conspicuous line patterns (Figs 11, 12).	***S. thutakanay***
–	Forewing with inconspicuous line patterns (Figs 5–10).	**2**
2	Hindwing with postmedial line close to outer margin (Figs 9, 10).	***S. mamanina***
–	Hindwing with postmedial line not close to outer margin (Figs 5–8).	***S. grata***

#### Sosxetra grata

Taxon classificationAnimaliaHymenopteraEurytomidae

Walker, 1862

AD3BE0F5-39D5-5D1C-AB28-5B6803C771AC

Figs 2, 3, 5–8, 13–15, 18, 22

Chaetoloma actinobola Felder, [1874]: pl. 99 fig. 20 (syn. Poole 1989). Type locality: R. Amazonas. [Unknown]. Note— Felder provided an illustration of a single male specimen of this species accompanied by a brief description comparing it to Sphinx japix Cramer (now Unzela japix (Cramer)) and noting its locality. The specimen was presumably lost during World War II.
Cereoctena agatha Möschler, 1880: 470–471, pl. 9 fig. 21 (syn. Poole, 1989). Type locality: Surinam. [MfNB]. Note— Möschler described this taxon with the assistance of Guenée, based on three specimens—two males and one female—collected from Paramaribo, and compared it with Ceroctena amynta (Cramer). Only two syntypes remain in the MfNB collection: one male and one female.

##### Neotype.

• ♂, Brazil Espirito Santo, Rio Lamego, XII-1951, A. Maller, Colln./ J. R. Neidhoefer Collection, MILWAUKEE PUBLIC MUSEUM/ MPM ENT101124/ DNA Voucher LEP-92924/ deposited in MPM. [examined]

##### Additional specimens examined.

(11 ♂, 3 ♀ MGCL; 3 ♂ MPM; 1 ♂, 1 ♀ MfNB; 6 ♂ JIM) **Brazil**: • Brazil, Rondonia, Cacaulandia, Fazenda Rancho Grande, road C-20 5 km. north & 3 km. west from Cacaulandia, lot 23 building area, at light, Coll. by D. L. Eiler, NOCTUIDAE, Rom-UM-715, FWL 20 mm deposited in MGCL (2 ♂); • Brazil, Rondonia, Cacaulandia, Fazenda Rancho Grande, road C-20 5 km. north & 3 km. west from Cacaulandia, lot 23 building area, at light, Coll. by D. L. Eiler, NOCTUIDAE, Rom-UM-715, FWL 20 mm/ DNA Voucher LEP-85585 (2 ♂). **Surinam**: • Surinam Prb. “nov. 74” / *Ceroctena agatha* Möshl:/ *Ceroctena agatha*/ Type. W.Z.B.g. X’X’X’.470./ MfN URI http://coll.mfn.-berlin.de/u/09f905 (1 ♂); Surinam Prb. “nov. 74” / *Ceroctena agatha* Möshl:/ *Ceroctena agatha*/ Type. W.Z.B.g. X’X’X’.470./ MfN URI http://coll.mfn.-berlin.de/u/09f720 (1 ♀). **Panama**: • Panama, R. P., Pinas Bay/ 4-24/28-66/ J.R. Neidhoefer Collection, MILWAUKEE PUBLIC MUSEUM/ MPM ENT101126/ DNA Voucher LEP-92925 (3 ♂). **Peru**: • Peru, Dep. Madre de Dios, Salvacion, Rio Alto de Madre de Dios, Manu – Park, ca. 500 m N.N. Dez. 1996, local people leg. EMEM 31.01.1997 (2 ♂). **Ecuador**: • Ecuador, Pichincha, Quito/ Chiriboga Km 33, 2650 m, 25 Apr. 1976, coll. N. Venedictoff (1 ♂); • Ecuador, Napo, Yasuni Nat. Pk., 20-IX–4-X–03, B/K- TLS Coll./ *Sosxetra grata*, det. E. Knudson/ MGCL Accession # 2019-5 E.C. Knudson, Knudson/Bordelon (1 ♂); • Ecuador, Pichincha, S. Dom., Tinalandia, 650 m, 4-V-1972, Coll Venedictoff/ Venedictoff colln., Allyn Museum, Acc. 1986-26 (1 ♀). **Colombia**: • Colombia, Cundinamarca, Guasca, El Chochal de Siecha, 3120 m, 28 Nov. 2019, coll. Jose I. Martinez (1 ♂). **Costa Rica**: • COSTA RICA, Guanacaste Prov. Hacienda Los Inocentes, 11°02'0.67", 85°30'14.29" elev. 350 m, Sept 1–3, 2005 *leg*. A. Sourakov (1 ♂); • COSTA RICA, Alajuela Province, Volcan Rincon de la Vieja, Las Bromelias, 640 m app. 10°52'N, 85°19'W, 13-16 September 2009, A. Sourakov *leg*/ A. Sourakov coll., MGCL Accession # 2009-28/ UF FLMNH MGCL 1126933 (2 ♀). **Guatemala**: • GUATEMALA, Guatemala, Puerta Parada, 1850 m, 17–23 VII 2017, Jose I. Martinez (1 ♂); • GUATEMALA, Sacatepéquez, Cerró Alux, 2230 m, 19-21 VII 2017, Jose I. Martinez (3 ♂); • GUATEMALA, Dept Izabal, Biotopo Chocon Machacas, 18-20-VI-2002, elev. 5 m at M.V. Light, J.C. Schuster & R.E. Woodruff (1 ♂). **Mexico**: • México, Yucatán, Tzucacab, Rancho Hobonil, UADY, 12-I-12, JI Martínez Coll./ UF FLMNH MGCL 1126100/ DNA voucher LEP-85588/ *Sosxetra grata* det. Jose I. Martinez, 2016 (1 ♂); • Mexico: STATE OF QUINTANA ROO, Xiatil/ H. L. KING, 28-X-67 Coll./ NOCTUIDAE Sosxetra grata/ DNA voucher LEP-85589 (1 ♂).

##### Diagnosis.

This species closely resembles *Sosxetra mamanina* in its bright pinkish-orange wings and faint forewing pattern. However, it can be distinguished by the presence of a whitish-yellow reniform spot and a distinctive diagonal mark near the forewing apex, the latter absent in *S. mamanina*. The forewings are bright pinkish-orange, featuring an elongated, broad, and faintly outlined “3”-like reniform spot. Five terminal dashes are present, with the first, third, and fifth broader than the others. The hindwings are similar in color but slightly brighter, except for the darker costal area, which bears a reduced pinkish lump covered with long whitish-brown hair-like scales. The postmedial line is pointed at vein M_1_, unlike in related species, where it is rounded. Four terminal dashes extend from M_1_ to CuA_2_, the last being barely visible. Forewing length in males ranges 39–41 mm. The palpus is marbled with bright orange, pink, and whitish-brown scales, while the underside is entirely whitish-brown. The thorax is bright pinkish-orange with whitish-brown markings medially and laterally, and whitish-brown ventrally. The abdomen is pale brownish-orange dorsally, with broad pinkish-orange dorsal tufts. The tuft on segment A1 is directed downward, while those from A2–A6 covering ¹/3 of the sternites, and the segment A7 bears a small tuft. The lateral and ventral surfaces are whitish-brown. The male genitalia are distinctive, characterized by short valvae with rounded apices and a short aedeagus ~3 × longer than wide. The vesica bears a broad dorsal meso-apical diverticulum, ~ ¹/3 the length of the vesica an autapomorphic feature for this species. Female genitalia remain unknown.

##### Immature stages.

***Eggs***. Unknown. ***Larva***. Late instar with pale green integument; head with black and white patterns; two broken black subdorsal lines surrounding white dots; a pair of yellow dorsal hardened plates in every tergite from A2-A7; wide verrucae with long white hair-like setae; wide white dorsal band (Fig. 23). ***Pre-pupa***. Integument changes to a dark reddish-pink; subdorsal line changes to white elongated dots; dorsal brown band A2-A7 including the area between the white spots; dorsal blurry grayish-brown band; pale gray hardened plates; verrucae with long dark gray hair-like setae. ***Pupa***. Unknown**. *Cocoon***. Thick walls covered by debris and plant residuals.

##### Genetic characterization.

Molecular data showed that *Sosxetra grata* (GenBank accession number: PQ406480–PQ406483; BOLD BIN number: BOLD:ADP8146, BOLD:AAA8259) is sister to *S. thutakanay* + *S. mamanina* with ~3.08–4.1% divergence (Fig. 1).

##### Etymology.

The name comes from the Latin word *gratus* that means beloved, dear, or grateful that might be based on this moth’s beauty.

##### Distribution.

**Figure 24.**
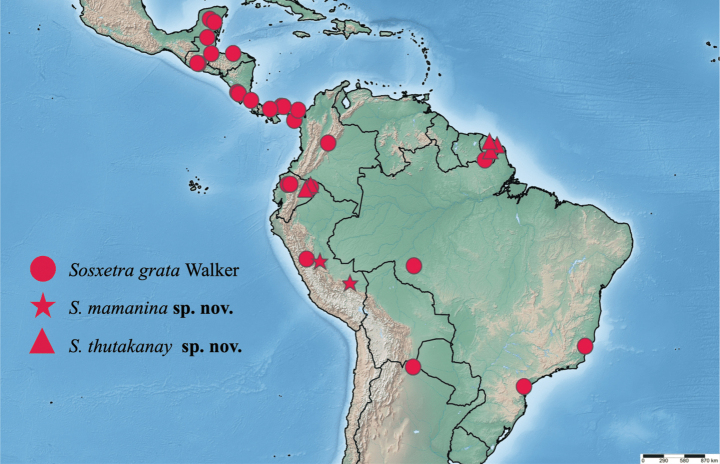
Distribution of examined specimens of the genera *Sosxetra*.

*Sosxetra grata* is the most widely distributed species of the genus, ranging from Mexico to Brazil (Fig. 24).

##### Remarks.

*Sosxetra grata* was found to represent a complex group with relatively low genetic divergence compared to other species. Due to limited specimen availability, we treat this group as a single species until further study. The only previously available information for this species was the original description by Walker (1862), with no known physical specimens available for examination. Access to such material is essential for comparative taxonomic and systematic work; therefore, we have designated a neotype to ensure accurate identification and provide a stable reference for future research. Although holotypes of Walker are typically housed at the NHMUK, Poole (1989) did not specify the repository for *S. grata*, and the NHMUK Data Portal currently lists the location of the type as unknown. We have redescribed the known species *Sosxetra grata* based on a neotype designated in this study. The newly designated neotype is the only known specimen that matches the diagnostic features described by Walker (1862) (Figs 5, 18).

#### Sosxetra mamanina

Taxon classificationAnimaliaHymenopteraEurytomidae

Martinez, Homziak & Castillo-Argaez sp. nov.

CD196ECB-EBD8-57E1-84AF-EF891BA01EF3

https://zoobank.org/C0CA6DA9-FF1D-40C8-8C67-DD16D7C321BF

Figs 9, 10, 19

##### Type material.

***Holotype***: • ♂, Peru, Dep. Madre de Dios, Salvacion, Rio Alto de Madre de Dios, Manu Park, 500 m, X–XI. 1996, local people leg. Colln. EMEM/ W. McGuire colln. MGCL Accession # 2008-43/ FLMNH-MGCL Specimen 167487/ DNA Voucher LEP-85587/ deposited in MGCL. ***Paratypes*** (4 ♂ MGCL): **Peru**: • Same collecting data as holotype (1 ♂); • Peru, Dep. Madre de Dios, Salvacion, Rio Alto de Madre de Dios, Manu – Park, ca. 500 m N.N. Dez. 1996, local people leg. EMEM 31.01.1997 (3 ♂).

##### Diagnosis.

Although *Sosxetra mamanina* is genetically closer to *S. thutakanay*, its external resemblance to *S. grata* is striking. Both species share a similar reduction of the antennal tufts and a short costal mid-lump covered with hair-like scales on the hindwing. However, *S. mamanina* can be readily distinguished from all other congeners by the position of the hindwing postmedial line, which lies unusually close to the outer margin, whereas in the remaining species it is situated farther inward (see Figs 5–12). Additionally, the terminal dashes in *S. mamanina* are larger than those observed in *S. grata*. The forewing reniform spot is narrow, resembling that of *S. grata*, but differs in the reduction or occasional absence of the black outline, which may be faint or indistinct. The male genitalia are also similar to those of *S. grata*, with a valva bearing a slightly rounded apex, yet they differ in the noticeably larger overall size of the valva.

##### Description.

***Head***. Palpus with marbled tufts of pink, purple, orange, and pale brown; frons pale brownish-orange; antenna brown with reduced pinkish-orange tufts. ***Thorax***. Dorsum pale brownish-orange; paler near wing bases; ventrum pale brownish-yellow. ***Leg***. Foreleg marbled pink, purple, orange, and pale brown, interspersed with white and gray; mid- and hindlegs concolorous with thorax ventrally. ***Wing***. Forewing length, male 40–42 mm; ground color pale brownish-orange; medial and postmedial lines faint, cream-colored; space between lines bright pink; interveinal lines pinkish-orange, darker near margin; thin white fold line present; reniform spot faint or absent; fringe orangish-yellow; six black terminal dashes; dash between R_4_ and R_5_ barely visible; apex unmarked; hindwing slightly brighter; costal and outer areas darker; cream postmedial line close to outer margin; costal margin with small bright pink lump, short hair-like scales; area between postmedial line and outer margin orangish-brown with purplish-blue spots; area between Sc+R_1_ and Rs with Հ-shaped line; fringe orangish-yellow with four large black dashes (M_1_–CuA_1_); posterior fringe pale yellowish-orange. ***Abdomen***. Dorsum orangish-yellow; ventrum paler, laterally brighter; brownish-orange dorsal tufts; A1 tuft broader with pink tips; A2–A5 tufts slender. ***Male genitalia***. Valva broad with pointed, slightly rounded apex; saccular process weakly rounded; juxta wide, ends swollen; uncus narrow, elongate, beak-like; saccus arrowhead-shaped; phallus with aedeagus 4½ × longer than wide; vesica slightly longer than aedeagus. ***Female genitalia***. Unknown.

##### Immature stages.

Life cycle and host plant remain unknown.

##### Genetic characterization.

Molecular data has shown that *Sosxetra mamanina* (GenBank accession number: PQ406478; BOLD BIN number: BOLD:AAZ7507) is sister to *S. thutakanay* with ~3.3% divergence (Fig. 1).

##### Etymology.

The name of this species is in reference to “*Mama Nina*” who is the “Mother of Fire” or “Fire Goddess” in the Incan culture (Villanueva-Sotomayor 2020).

##### Distribution.

This species is only known from south-central Peru (Fig. 24).

##### Remarks.

Holotype and paratypes are in perfect condition (Figs 9, 10).

#### Sosxetra thutakanay

Taxon classificationAnimaliaHymenopteraEurytomidae

Martinez, Homziak & Castillo-Argaez sp. nov.

18BED582-556C-544B-A622-72DB30DA8FB5

https://zoobank.org/5B1FAE92-C815-44E8-B46C-4628F3D03115

Figs 11, 12, 20

##### Type material.

***Holotype***: • ♂, Ecuador, Napo, Yasuni Nat. Pk., 20-IX–4-X–03, B/K- TLS Coll./ Sample Number 7463, COI-09/ MGCL Accession # 2019-5 E.C. Knudson, Knudson/Bordelon/ UF FLMNH MGCL 1126522/ DNA Voucher LEP-85592. Deposited in MGCL. ***Paratype*** (1 ♂ MGCL): **French Guiana**: • French Guiana, Kaw Camp Caiman, Kaw Mt., 20-25 October 2017, 300 m. J. B. Heppner/ DNA Voucher LEP-85587 (1 ♂).

##### Additional specimens examined.

(2 ♂ MGCL) **Ecuador**: • Same collecting data as holotype and paratype.

##### Diagnosis.

*Sosxetra thutakanay* can be readily distinguished from other species in the genus by its distinctive wing pattern. The forewing bears a broad white band along the fold, while the hindwing displays a unique knife-shaped marking between veins Rs and M_1_, formed by whitish-purple scales, features not present in any other congeners. In addition, the male genitalia are diagnostic, characterized by a valva with a distinctly beak-like apex, a unique trait that separates this species from all others in the *Sosxetra* genus.

##### Description.

***Head***. Palpus marbled brown and dark brown, ventrally divided whitish-brown and brown; frons brownish-orange, darker than thorax; antenna brown with marbled brown and dark brown tufts. ***Thorax***. Dorsum reddish-brown, paler medially; ventrum whitish-brown; patagium concolorous dorsally, slightly darker. ***Leg***. Foreleg marbled as in palpus and patagium; mid- and hindlegs concolorous with thorax ventrally. ***Wing***. Forewing length, male 35–38 mm; forewing reddish-brown dorsally, whitish-brown ventrally; area between medial and postmedial lines bright orangish-pink; broad white band along wing fold, nearly covering CuA2 and Sc+R1 between medial and postmedial lines.; reniform spot large, bean-shaped; reniform spot crossed by two dark brown lines basally in R_3_, R_4_, R_5_; veins and interveinal lines dark brown, separated by orangish-pink bands; fringe brownish-yellow with six wide black terminal dashes; apex marked by narrow, sinuous brownish-yellow curve; hindwing similar in coloration to forewing; discal area matching orangish-pink tone of forewing; postmedial line bright orangish-pink; costal margin with long brownish-yellow hair-like scales; dark reddish-brown mid-lump; area between postmedial line and outer margin dark brown; regions between Sc+R_1_–M_1_ dark orange with whitish-purple scales; space between Rs–M_1_ with knife-tip shaped whitish-purple mark; fringe brownish-yellow with four black dashes between M_1_–CuA_1_, broader between M_1_–M_3_; posterior fringe whitish-brown, covering ~¹/3 × of wing. ***Abdomen***. Entirely whitish-brown with reddish-brown tufts; last three sternites darker; A1 with broad tuft, A2 smaller, A3–A7 tiny; genital area with long brown scales. ***Male genitalia***. Valva broad, apex beak-like; sacculus rounded on outer margin; juxta straight, with minute medial notch; uncus short, beak-shaped; saccus V-shaped, lower side rounded; phallus with aedeagus 8½ × longer than wide; vesica simple, subequal in length to aedeagus. ***Female genitalia***. Unknown.

##### Immature stages.

Life cycle and host plant association in this species remain unknown.

##### Genetic characterization.

The DNA barcode placed *Sosxetra thutakanay* (GenBank accession number: PQ406479; BOLD BIN number: BOLD:AAJ4009) sister to *S. mamanina* with ~3.3% divergence (Fig. 1).

##### Etymology.

The species name is a combination of two Quechuan words *thuta* meaning moth and *kanay* which can be translated as ember (Laime-Ajacopa 2007).

##### Distribution.

The specimens collected are from Ecuador and French Guiana (Fig. 24).

##### Remarks.

Specimens from Ecuador (Fig. 11) are slightly different from those of French Guiana (Fig. 12), but DNA showed 0.8% divergence which is why we placed them in the same species, but this needs examination of more specimens and further information, especially immature stages. Among the three Ecuadorian specimens, only one was well-preserved and selected as the neotype. Although the other two were severely damaged, they were still identifiable based on morphology; however, DNA barcoding was not performed on these specimens.

#### Covellana

Taxon classificationAnimaliaLepidopteraNoctuidae

Martinez, Homziak, Plotkin & Castillo-Argaez gen. nov.

51A7BDB4-763E-5D6F-911B-DA06617DE0F4

https://zoobank.org/337DF7D2-A976-49A8-A12B-F9A8A60D5B07

##### Gender.

Feminine.

##### Type species.

*Covellana niomalan* sp. nov. by present designation.

##### Included species.

The genus *Covellana* gen. nov. consists of a single species: *Covellana niomalan* sp. nov. This species was previously misidentified as *Sosxetra grata*, but our detailed morphological examination allowed us to recognize a new genus closely related to *Desmoloma*.

##### Diagnosis.

*Covellana* appears closely related to the genera *Sosxetra* and *Desmoloma* based on morphological evidence and is currently known from four well-preserved male specimens. However, its wing pattern differs noticeably from its relatives. The markings in *Sosxetra* are generally more distinct than in *Covellana* and *Desmoloma*. Unlike *Sosxetra*, *Covellana* lacks the broad, bright reniform spot that characterizes the former, and instead exhibits antemedial and subterminal lines resembling those of *Desmoloma*. The hindwing also shows significant differences among the three genera. *Covellana* lacks both the terminal dashes and the bright posterior fringe typical of *Sosxetra*, but possesses three small lumps along the costal margin, a feature more consistent with *Desmoloma*. The most important diagnostic traits of *Covellana* are found in the male genitalia. The valvae are unfused but extremely delicate and difficult to separate without causing damage. The genus is best distinguished by the structure of the phallus, which bears a vesica with a basal diverticulum and an aedeagus featuring an additional diverticulum in the middle. The female of the genus remains unknown.

##### Description.

***Head***. Palpus long, brown with large tufts; whitish-brown on last segment; frons concolorous with thorax; antenna bipectinate; basal ¹/6 and apical ¹/6 without pectinations. ***Thorax***. Dorsum darker medially; ventrum pale brown; patagium reduced, same color as thorax. ***Leg***. Pale brown; foreleg with long tufts matching thoracic color; mid- and hindlegs concolorous with thorax ventrally. ***Wing***. Forewing length, male 34–39 mm; ground color dark reddish-brown to brown; diffuse black transverse lines; basal line broken, paler; antemedial, postmedial, and subterminal lines well-defined; orbicular spot faint; reniform spot broad, curved, sometimes “3”-shaped; fringe bright yellow; black terminal dashes at vein ends; hindwing similar in color to forewing; slightly paler along costa; costa with three small lumps; postmedial and subterminal lines distinct but interrupted medially; distal area with broad dark shade; outer fringe yellow from Sc+R_1_–CuA_1_, remainder brown; terminal dashes as in forewing; posterior fringe brown. ***Abdomen***. Dorsum dark brown, ventrum whitish-brown. ***Male genitalia***. Valvae Dutch clog-like, not widely open; tegumen narrow with slender median lobes; juxta rectangular with swollen lower spots; saccus narrow, V-shaped; phallus short; aedeagus broad with a diverticulum; vesica elongate, bearing basal diverticulum. ***Female genitalia***. Unknown.

##### Genetic characterization.

Comparison of the DNA barcode for *C. niomalan* to the 25,379 noctuoid records in the Barcode of Life Data System v4 revealed that *Covellana* was genetically related to species of the genus *Desmoloma* (Fig. 1). This relationship is further supported by similarities in external morphology and resting position.

##### Etymology.

*Covellana* is dedicated to our mentor and friend, Dr. Charles V. Covell Jr., an American entomologist whose passion for butterflies and moths began at the age of 13. During the past 70 years, he has built an extraordinary legacy within the entomology community both nationally and internationally.

##### Immature stages.

Life cycle and host plant association in this species remain unknown.

##### Biology.

Unknown.

#### Covellana niomalan

Taxon classificationAnimaliaLepidopteraNoctuidae

Martinez, Homziak, Plotkin & Castillo-Argaez sp. nov.

552161CB-978D-5376-9162-899698E4B62A

https://zoobank.org/36C35CE8-6049-4E46-BA9D-B4A57F24ACCD

Figs 4, 16, 17, 21

##### Type material.

***Holotype***: • ♂, PANAMA., CANAL ZONE, Dump 9., 29-IX 1982, D. H. Habeck, MV and Black light/ DNA Voucher LEP-94957. deposited in MGCL. ***Paratypes*** (4 ♂ MGCL): **Panama**: • Same collecting data as holotype/ DNA Voucher LEP-94958 (1 ♂); PANAMA., Middle Chagras, 21-IX-1982, D. H. Habeck, MV and Black light (1 ♂). **Peru**: • Peru, Dep. Madre de Dios, Salvacion, Rio Alto de Madre de Dios, Manu Park, 500 m, X–XI. 1996, local people leg. Colln. EMEM/ W. McGuire colln. MGCL Accession # 2008-43/ FLMNH-MGCL Specimen 168181/ DNA Voucher LEP-94955 (2 ♂).

##### Diagnosis.

*Covellana niomalan* can be superficially confused with species of *Sosxetra* due to their similar external appearance and body coloration. However, it can be distinguished by several key features. Unlike *Sosxetra*, *C. niomalan* lacks the terminal dashes on the wings. The forewing of *C. niomalan* bears a dark reniform spot, whereas *Sosxetra* species typically have white reniform spots. The hindwing of *C. niomalan* presents three yellow lumps along the costal margin, in contrast to the single white lump found in *Sosxetra*. The male genitalia are also diagnostic, with the aedeagus exhibiting a narrow diverticulum, a feature unique to this species.

##### Description.

***Head***. Palpus brown, tips whitish-brown; frons orangish-brown; antenna pale brown; scape with small pale pinkish-brown tufts. ***Thorax***. Dorsum orangish-brown, darker medially; ventrum whitish-brown with black shading. ***Leg***. Foreleg with orangish-brown tufts; mid- and hindlegs concolorous with thorax ventrally. ***Wing***. Forewing length, male 33–35 mm; ground color dark reddish-brown; diffuse black lines; basal to medial area orange; postmedial line curved at wide C-shaped black reniform spot; inner margin paler; outer margin with thin bright yellow fringe; no terminal dashes; hindwing darker, similar to forewing; posterior area much darker; transverse lines well-defined; postmedial line curved, spiky; subterminal line faint; discal shade wide, reddish-purple; costal fringe with three yellow lumps bearing long pale yellow hair-like scales; outer fringe bright yellow; pointed terminal dashes at vein ends; posterior fringe pale reddish-brown. ***Abdomen***. Dorsum pale reddish-brown, ventrum pale brown; A1 tuft broad, divided orangish-brown area ²/3 × larger than white area. ***Male genitalia***. Valva Dutch clog-like, apex wide and rounded; saccular process smoothly angled; juxta with single lower swollen spot; uncus scorpion-sting shaped, swollen basally; saccus V-shaped; phallus with aedeagus 2½ × longer than wide, narrow diverticulum; vesica ¼ × longer than aedeagus. ***Female genitalia***. Unknown.

##### Immature stages.

Life cycle and host plant association remain unknown.

##### Genetic characterization.

The DNA barcode obtained for *C. niomalan* exhibits a genetic affinity to *Desmoloma modesta*, compared to other *Desmoloma* species, with an approximate divergence of 6.1% (GenBank accession number: PV077143) (Fig. 1).

##### Etymology.

The species epithet is derived from the Guaymí word *niö*, which means fire and *malan* meaning butterfly or moth (Alphonse 1956).

##### Distribution.

**Figure 25.**
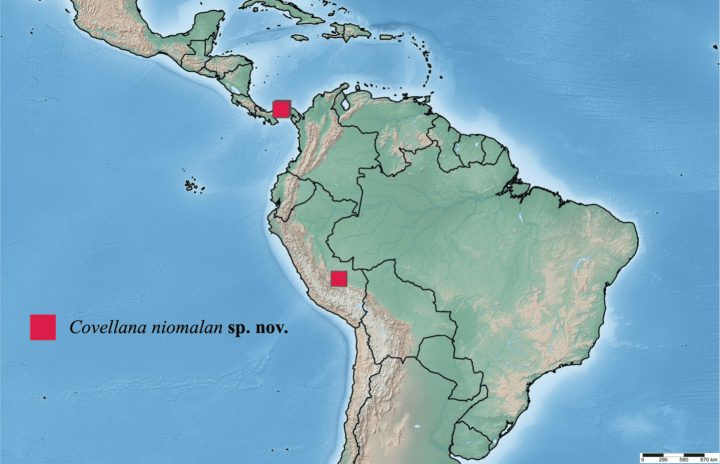
Distribution of examined specimens of the genera *Covellana*.

This species is currently known only from two populations, one in Panama and the other in Peru (Fig. 25).

##### Remarks.

Although the Panamanian (Fig. 16) and Peruvian (Fig. 17) populations exhibited some morphological differences, we were unable to obtain DNA barcodes of the Peruvian population for comparison. Therefore, we have chosen to treat both populations as a single species. The holotype and paratypes were in excellent condition, although one of the paratypes had a detached abdomen, which was used for genitalia preparation. While *Desmoloma modesta* is the species most closely related to *Covellana niomalan*, only a few *Desmoloma* species are available in the Barcode of Life Data System v4 (https://v4.boldsystems.org/), indicating the need for further investigation.

## Discussion

The species *Sosxetra grata* Walker was the only species known in this genus for many decades due the other species synonymized within it (Poole 1989). This phenomenon is common with cryptic species where it is very difficult to determine or separate these species (Schmidt and Sperling 2008). Also worth noting that the vast majority of the hyperdiverse Neotropical Noctuoidea remain without modern taxonomic revision, and that many if not most species remain undescribed. Now with advances in the molecular era taxonomists and systematists are starting to use genetic or even genomic data to resolve long standing taxonomic issues (Kawahara et al. 2023). Unfortunately, some people are supplanting the use of morphology with DNA data alone, creating more taxonomic problems than solutions (Sharkey et al. 2021). These kinds of studies do not consider genetic bias such as hybridization or introgression, which is common in insects (Arce-Valdés and Sánchez-Guillén 2022). Thankfully, many other researchers have begun to combine morphological and molecular data to build more stable classification (Schmidt and Anweiler 2010; Martinez 2020).

**Figures 26, 27.**
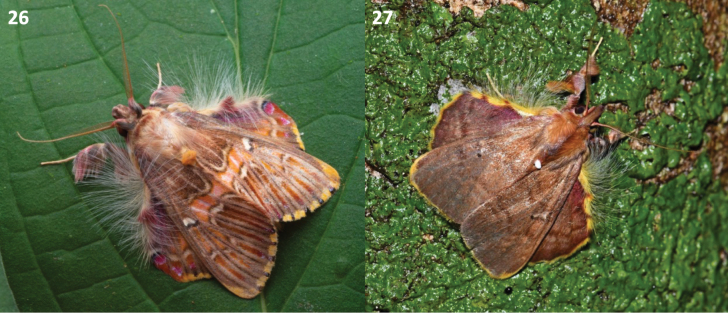
Resting position of *Sosxetra* and *Covellana*. **26**. *S. grata*, ♂, Cayo District, Belize photo by Thomas Shahan; **27**. *Covellana niomalan* Barro Colorado Island, Panama photo by Maxim Larrivée.

Herein, we corroborated the relationship between *Sosxetra* and *Ceroctena* proposed by Zahiri et al. (2013) and Keegan et al. (2021) and further corroborate the evolutionary convergence of the resting position with Lymantriinae (Figs 26, 27). This is also emphasized by the host plant association where both genera have been recorded feeding on species of the family Meliaceae that include some at-risk plants such as *Guarea luxii* C. DC. (Janzen and Hallwachs 2009; IUCN 2025; JIM, per. obs.). This association with host plants of conservation interest in addition with the charisma and beauty of the members of *Sosxetra* demonstrate the potential of this genus to serve as a flagship taxon and/or a target for conservation purposes. Additionally, we were able to delineate the new genus *Covellana*, previously confused as variation within *Sosxetra* in museum collections worldwide as well as online platforms. Furthermore, our analysis suggests that *Covellana* is more closely related to *Desmoloma modesta* (Dognin 1923) than to *Sosxetra*. *Ceroctena*, *Covellana*, *Desmoloma modesta*, and *Sosxetra* are linked by the presence of fused valvae. For further clarification, the Cornell University Insect Collection (CUIC) website (https://cuic.entomology.cornell.edu/) features a mounted genitalia of *Betusa* Walker (now *Ceroctena*) (“No, 1088, *Betusa*, DATE. Dec. 21, 1938, J.G. Franclemont”). This specimen shows open valvae, which distort the genital features when compared to those with fused valvae (JIM unpubl. data).

Our phylogenetic analyses strongly support the separation of *S. mamanina* and *S. thutakanay* as distinct species from *S. grata* (UFBoot2 ≥ 100/SH-aLRT ≥ 93.5). These findings are further corroborated by morphological evidence, which reinforces the species-level distinctions observed in the molecular data. On the other hand, *S. grata* was found to be a single species without good evidence of other species within it due to the low genetic divergence (1.2–2.3%) compared to the other species, and moderate phylogenetic support (UFBoot2 = 91 / SH-aLRT = 91.8). However, due to the lack of available specimens for morphological comparison in some clades, we chose to treat the group as a single species. In contrast, *S. mamanina* and *S. thutakanay* also form a clade but exhibit a higher genetic divergence of ~3.3–3.5%, which is supported by relatively low phylogenetic values (UFBoot2 = 59/SH-aLRT = 60.3). This pattern in our results is consistent with known cases of mtDNA introgression or introgressive hybridization, a phenomenon documented in other noctuoid groups (Anweiler 2009; Otim et al. 2018; Martinez 2020; Schmidt and Anweiler 2020). Moreover, it has long been recognized that DNA barcodes (COI) often have limited resolution in distinguishing recently diverged sister species, complicating the identification of cryptic species (Tavares and Baker 2008; Struck et al. 2018; Ranasinghe et al. 2022). Given the moderate geographic overlap among individuals of the *S. grata* complex, this case may reflect a scenario of recent sympatric speciation. In the early stages of divergence, populations may become reproductively isolated due to factors such as differences in genital morphology or ecological behavior, while still remaining genetically similar and often lacking clear barcode gaps. These conditions make species delimitation particularly challenging (van Velzen et al. 2012; Schmidt et al. 2013). However, this hypothesis cannot be fully corroborated with our current dataset.

Finally, despite the species *Sosxetra grata* has been commonly known as “Walker’s moth”; this name was proposed when this genus was considered monotypic. However, now that there is more than one species, we propose to name the group “comose flame moths” based on the fact that these species have very bright orange, red, and yellow colors, while for *Covellana* species we propose the name “comose fire moths” in order to make reference to similar features between both genera.

## Supplementary Material

XML Treatment for Sosxetra

XML Treatment for Sosxetra grata

XML Treatment for Sosxetra mamanina

XML Treatment for Sosxetra thutakanay

XML Treatment for Covellana

XML Treatment for Covellana niomalan

## Additional information

### Use of AI

No use of AI was reported.

### Author ORCIDs

Jose I. Martinez https://orcid.org/0000-0003-4368-2729

Nicholas T. Homziak https://orcid.org/0000-0002-8855-1303

Taylor L. Pierson https://orcid.org/0009-0005-2833-1260

Rhys J. L. Campo https://orcid.org/0009-0008-7346-2683

David M. Plotkin https://orcid.org/0000-0002-2339-655X

Raiza J. Castillo-Argaez https://orcid.org/0000-0001-9344-1494

### Data availability

All of the data that support the findings of this study are available in the main text or Supplementary Information.

## Supplementary materials

Supplementary material 1 Phylogenetics Data type pdf Explanation note Gene tree inferred employing Maximum likelihood in IQ-TREE based on the mitochondrial cytochrome oxidase (COI) marker and rooted to *Dyops cuprescens*. https://binary.pensoft.net/file/1533729This dataset is made available under the Open Database License (http://opendatacommons.org/licenses/odbl/1.0/). The Open Database License (ODbL) is a license agreement intended to allow users to freely share, modify, and use this Dataset while maintaining this same freedom for others, provided that the original source and author(s) are credited. Jose I. Martinez, Nicholas T. Homziak, Taylor Pierson, Rhys Campo, David M. Plotkin, Raiza J. Castillo-Argaez

Supplementary material 2 Information data Data type xlsx Explanation note Sequences information employed in this study available at the GenBank (https://www.ncbi.nlm.nih.gov/genbank/) and the Barcode of Life Data System v4 (https://v4.boldsystems.org/). https://binary.pensoft.net/file/1533730This dataset is made available under the Open Database License (http://opendatacommons.org/licenses/odbl/1.0/). The Open Database License (ODbL) is a license agreement intended to allow users to freely share, modify, and use this Dataset while maintaining this same freedom for others, provided that the original source and author(s) are credited. Jose I. Martinez, Nicholas T. Homziak, Taylor Pierson, Rhys Campo, David M. Plotkin, Raiza J. Castillo-Argaez

Supplementary material 3 Bioinformatics Data type tree Explanation note Tree file in NEWICK format correlated with the phylogenetic tree in Fig. 1 and Suppl. material 1. https://binary.pensoft.net/file/1533731This dataset is made available under the Open Database License (http://opendatacommons.org/licenses/odbl/1.0/). The Open Database License (ODbL) is a license agreement intended to allow users to freely share, modify, and use this Dataset while maintaining this same freedom for others, provided that the original source and author(s) are credited. Jose I. Martinez, Nicholas T. Homziak, Taylor Pierson, Rhys Campo, David M. Plotkin, Raiza J. Castillo-Argaez
