# Supplementary figures and images for: Revision of the comose flame moths of the genus Sosxetra Walker (Noctuidae, Dyopsinae), with descriptions of a new genus and three new species
Source: Zookeys. 2026 Feb 6;1268:227–48. doi: 10.3897/zookeys.1268.138260 (PMC12905589; doi:10.3897/zookeys.1268.138260)

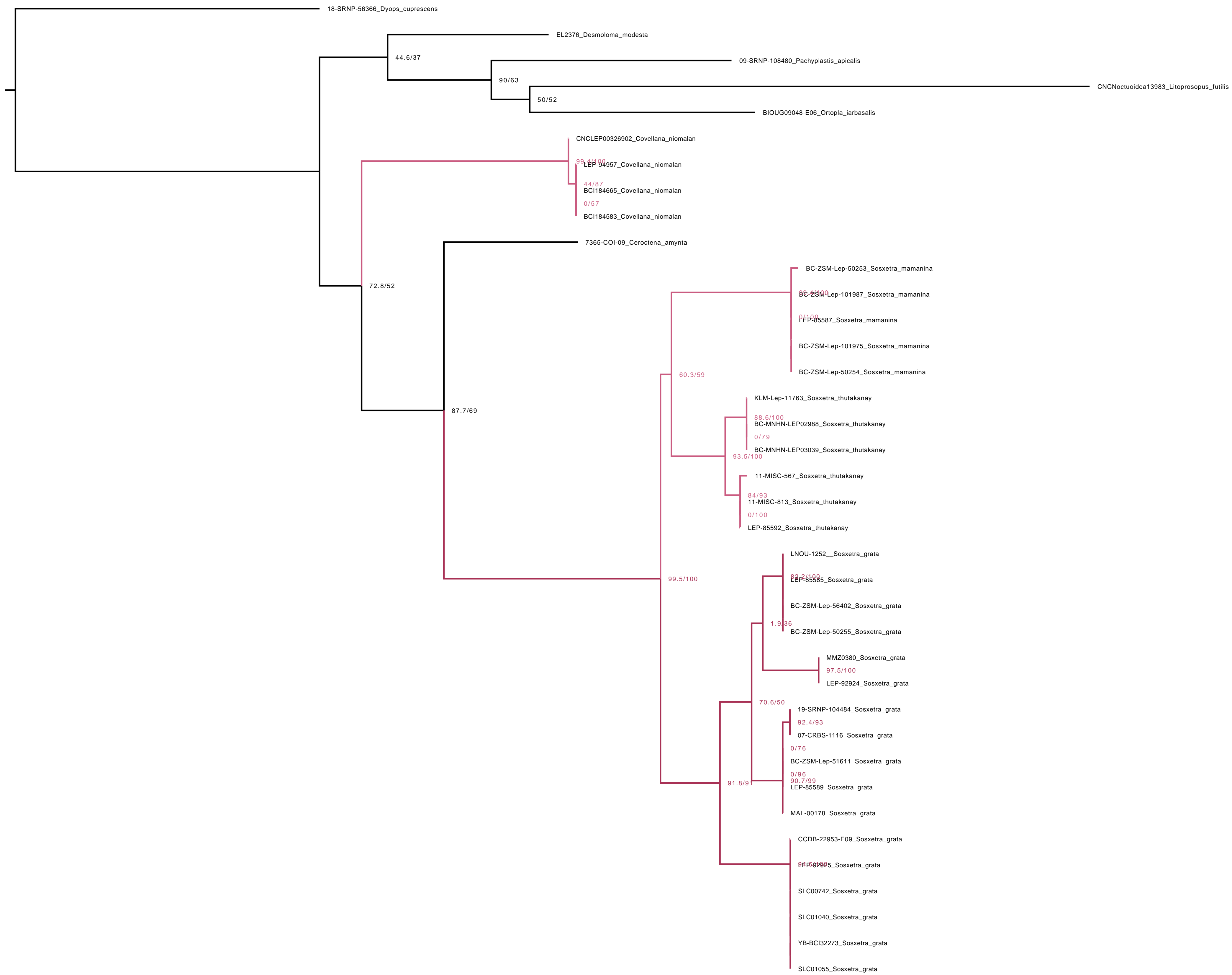

0.02

Supplement: Supplementary material 1 — Phylogenetics [file zookeys-1268-227_article-138260__-s001.pdf]
